# Implicit and explicit learning of socio-emotional information in a dynamic interaction with a virtual avatar

**DOI:** 10.1007/s00426-022-01709-4

**Published:** 2022-08-29

**Authors:** Andrei R. Costea, Răzvan Jurchiș, Laura Visu-Petra, Axel Cleeremans, Elisbeth Norman, Adrian Opre

**Affiliations:** 1 Cognitive Psychology Laboratory, Department of Psychology, Babeș-Bolyai University, Cluj-Napoca, Romania; 2 Department of Socio-Human Research, Romanian Academy, Cluj-Napoca Branch, Cluj-Napoca, Romania; 3 Research in Individual Differences and Legal Psychology (RIDDLE) Laboratory, Department of Psychology, Babeș-Bolyai University, Cluj-Napoca, Romania; 4 Consciousness, Cognition and Computation Group (CO3), Center for Research in Cognition and Neuroscience (CRCN), ULB Neuroscience Institute (UNI), Université Libre de Bruxelles, Brussels, Belgium; 5 Department of Psychosocial Science, Faculty of Psychology, University of Bergen, Bergen, Norway

## Abstract

Implicit learning (IL) deals with the non-conscious acquisition of structural regularities from the environment. IL is often deemed essential for acquiring regularities followed by social stimuli (e.g., other persons’ behavior), hence is hypothesized to play a role in typical social functioning. However, our understanding of how this process might operate in social contexts is limited for two main reasons. First, while IL is highly sensitive to the characteristics of the surface stimuli upon which it operates, most IL studies have used surface stimuli with limited social validity (e.g., letters, symbols, etc.). Second, while the social environment is dynamic (i.e., our behaviors and reactions influence those of our social partners and vice-versa), the bulk of IL research employed noninteractive paradigms. Using a novel task, we examine whether IL is involved in the acquisition of regularities from a dynamic interaction with a realistic real-life-like agent. Participants (*N* = 115) interacted with a cinematic avatar that displayed different facial expressions. Their task was to regulate the avatar’s expression to a specified level. Unbeknownst to them, an equation mediated the relationship between their responses and the avatar’s expressions. Learning occurred in the task, as participants gradually increased their ability to bring the avatar in the target state. Subjective measures of awareness revealed that participants acquired both implicit and explicit knowledge from the task. This is the first study to show that IL operates in interactive situations upon socially relevant surface stimuli, facilitating future investigations of the role that IL plays in (a)typical social functioning.

**Supplementary Information:**

The online version contains supplementary material available at 10.1007/s00426-022-01709-4.

## Introduction

One of the biggest contributors to the evolution of humankind is our ability to swiftly function as part of complex social systems. Recent perspectives suggest that humans acquire the necessary skills and knowledge to effectively interact within such systems through unconscious learning processes (e.g., Lieberman, 2000; Norman & Price, 2012). Unconscious or implicit learning (IL; see Cleeremans et al., 1998, for a review), as opposed to its explicit counterpart, refers to the acquisition of complex regularities in the absence of a conscious intention to learn and without full conscious awareness of the acquired knowledge (Reber, 1967).

Several lines of research have found empirical evidence for the involvement of IL in social functioning. For instance, in the hidden covariation detection task, researchers have found that participants are able to implicitly learn an arbitrary covariation between a physical feature (e.g., short vs. long hair) and personality features (e.g., kindness), and that the judgement of new avatars is influenced by the learned covariation (e.g., Ivanchei et al., 2019; Lewicki, 1986). Similarly, Norman and Price (2012) have shown that participants are able to implicitly learn a grammar that determines the order of body movements executed by another person. Strachan et al. (2019) showed that statistical regularities regarding other people’s behavior—specifically, whether a person provides mostly facilitative or misleading information—can also be learned unconsciously. Colloquially, one can evaluate the affective state of familiar persons even without being aware of the evidence that supports one’s evaluation (e.g., “I know my friend is upset, but I cannot explain how I know—I just know”).

Due to the proposed centrality of this process for adaptive social functioning, impairments in IL have been investigated, with mixed results, as etiopathogenetic mechanisms in several disorders characterized by impairments in social functioning. For brevity, we only mention the equivocal findings obtained in autism (e.g., Brown et al., 2010; Gordon & Stark, 2007; Nemeth et al., 2010; Zwart et al., 2017) and depression (e.g., Borbély-Ipkovich et al., 2014; Exner et al., 2009; Janacsek et al., 2018).

### Limitations of the extant research investigating IL in relation to the social domain

The vast amount of evidence for the existence of IL has been obtained using non-social stimuli and tasks, such as the Artificial Grammar Learning task (AGL; Reber, 1967) or the Serial Reaction Time task (SRTT; Cleeremans & McClelland, 1991; Nissen & Bullemer, 1987). In these paradigms, participants learn regularities that structure nonsocial stimuli such as letter strings (in AGL) or spatial locations (in the SRTT) and are generally considered to provide robust evidence for IL effects (e.g., Dienes & Seth, 2018; but see Shanks, 2004, 2010). In contrast, there is very limited direct evidence that IL can operate upon socially relevant stimuli, the evidence being restricted to the aforementioned studies (e.g., Norman & Price, 2012) and few others (but see Hendrickx et al., 1997). Hence, the claims that IL could be involved in social functioning does not primarily rest on studies using social stimuli, but rather on the assumption that IL is a general process which unfolds in the same way, regardless of the specific stimuli on which it operates (e.g., Pothos et al., 2006)—e.g., if IL operates on letter strings, it might operate roughly similarly with social stimuli. Consequently, for instance, the studies that attempt to test whether social functioning deficits in some disorders could be caused by deficits in implicit learning use such nonsocial tasks (AGL, SRTT) and assume to test a “general” implicit learning capacity (e.g., Exner et al., 2009; Naismith et al., 2006, 2010; Nemeth et al., 2010; Rathus et al., 1994; Zwart et al., 2018).

The assumption that IL is a general capacity is, however, at odds with a consistent corpus of empirical findings: the extant literature on the boundary conditions for IL clearly shows that we cannot implicitly extract regularities from all categories of stimuli to the same extent. For instance, participants automatically learn a task-irrelevant artificial grammar when it is instantiated by evolutionary relevant stimuli (human faces), but not when it is instantiated by evolutionary irrelevant stimuli (buildings) (Eitam et al., 2014); see also Dienes and Altmann (1997), Jimenez et al. (2020), or Scott and Dienes (2010), for further evidence for the dependency of IL on the characteristics of the surface stimuli. Closer to the social domain, Ziori and Dienes (2015) found that an artificial grammar was learned less when it structured sequences of faces (56% classification accuracy), compared to the same grammar that structured letter strings (64% accuracy; Dienes & Scott, 2005). In a direct comparison, Norman and Price (2012) found less learning of an artificial grammar that structured sequences of body postures compared to the same grammar when it structured letter sequences (53% vs 58% classification accuracy). Further evidence comes the related field of statistical language learning, which is often assumed to occur, partially, implicitly: Li et al. (2022) have found that young adults with a high level of autistic traits are able to extract statistical regularities from non-social auditory input (pure tones), but not from socially relevant auditory input (Chinese disyllables), bringing further support for the stimulus-dependent operation of implicit/statistical learning. In sum, there is robust evidence that the surface stimuli influence the amount of learning in typical implicit learning tasks. There is also emerging evidence that, at least in the AGL task, learning is lower with more complex, social stimuli, compared to the typical version that uses letters. Accordingly, given that IL functions differently with different types of stimuli, it may be unwarranted to make inferences regarding the role played by IL in the social domain based on studies that use non-social surface stimuli (see Norman & Price, 2012; Jimenez et al., 2020, for thorough analyses of this problem). Moreover, given the emerging evidence that complex social stimuli are learned less well than simpler nonsocial stimuli (e.g., Ziori & Dienes, 2015), a legitimate concern is whether there is any learning for even more ecologically relevant social stimuli than those already used, such as dynamic facial emotional expression.

A different limitation is that the few existing studies that have employed more socially relevant stimuli have used experimental paradigms in which participants are exposed to social contingencies in a relatively passive manner. For instance, in the hidden covariation detection task, participants are exposed to avatars that instantiate contingencies between physical and psychological features, but there is no change in the participants’ behavior as a result of the avatar’s behavior or vice versa. In contrast, as we will clarify in the subsequent section, the central feature of our social life is its deeply interactive, dynamical nature.

### Characteristics of the information exchange loops

We start from the observation that in both real-life and experimental environments, information is being exchanged in loops. Specifically, in most cases of real-life interaction, an individual’s behavior determines or encourages a response from the social environment; further, the individual typically reacts again to the social environment’s response—thus perpetuating a loop of informational exchange. Similarly, in experimental contexts, a participant’s behavior determines a response from the research paradigm (e.g., advancing to the next stimulus) to which the participant typically reacts again, thereby perpetuating a loop of informational exchange.

However, the loops through which information is exchanged in most IL tasks are fundamentally different from those in which information is exchanged in social environments. Specifically, in the real-world environment, if an individual behaves in different manners, s/he should expect different responses—i.e., information is being exchanged via feedback-driven interactive loops (Becchio et al., 2010). By contrast, in most IL paradigms, participants respond to overly complex, predefined sequences of stimuli which, crucially, do not adapt in reaction to their responses—i.e., information is being exchanged via noninteractive loops. For instance, in the acquisition phase of the AGL participants are exposed to a predetermined list of letter strings; there is no modulation in the behavior of the task or of the stimuli as a consequence of participant’s behavior. The same principle applies to the SRTT and to the hidden covariation detection task.

Here, we emphasize that an instrument that aims to assess the role of IL in social interactions—besides using socially relevant surface stimuli—should also simulate the dynamic, feedback-driven manner in which information is being exchanged in such environments. One example of such a method is the classic *Dynamic Systems Control* task (DSC; Berry & Broadbent, 1984)—which will be detailed in the paragraphs below.

In the DSC, participants gradually learn to control the value of a dynamic system’s output variable by manipulating the value of an input variable. For instance, in the Sugar Production condition of their seminal paper, Berry and Broadbent (1984) asked participants to reach and maintain a certain output of a small sugar factory. Participants could achieve their task simply by manipulating the number of employed workers in any given trial. Unbeknownst to the participants, however, the relationship between their inputs and the amount of sugar production was mediated by a counterintuitive equation such that the same input from the participant determined different outputs depending on the previous state of the system. In other words, the system interacted with the participants, by adapting its responses according to participants’ successive inputs, thereby implementing an interactive loop of informational exchange. Berry and Broadbent (1984) found that participants improved their performance maintaining the desired output as the task progressed, suggesting that learning occurred. Furthermore, as assessed by a written post experimental questionnaire, the authors found that the performance improvement was not associated with reportable knowledge of the underlying rule, suggesting that learning had been implicit. Congruent results were also obtained by several other investigations (e.g., Berry & Broadbent, 1995; Dienes & Fahey, 1995; Fahey & Dienes, 1998).

Berry and Broadbent (1984) also attempted to adapt their Sugar Production task to assess the role of IL in social interactions. For this purpose, in the Personal Interaction condition of their experiment, the authors told the participants that they would be interacting with a virtual person named Clegg. Participants were further informed that their task was to keep Clegg’s mood at a prespecified level. Participants could enter their inputs via a keyboard and Clegg’s responses were presented on a display as linguistic labels (i.e., text vignettes). The following adjectives were used both as Clegg’s possible moods and as participants’ response options: “Very Rude, Rude, Very Cool, Cool, Indifferent, Polite, Very Polite, Friendly, Very Friendly, Affectionate, Very Affectionate or, Loving”. Each of the adjectives was assigned a numerical value from 1 to 12. Unbeknownst to the participants, the relationship between their inputs and Clegg’s mood was mediated by the same equation as that used in the Sugar Production condition. Berry and Broadbent (1984) found that participants improved their performance to keep Clegg’s mood on the target state as the task progressed, suggesting that learning occurred in the task. As in the Sugar Production condition, learning was also found to be mostly implicit.

Importantly, we suggest that while the Personal Interaction task implements interactive loops of informational exchange between the participants and the task, its use of linguistic labels as surface stimuli keeps it abstract in a way that severely limits its relevance to the social domain.

### The present study

While we consider that the existing studies on the involvement of IL in our social functioning provide encouraging starting results, we also think that their relevance for the social world is restricted by two main factors: (a) the artificial nature of the surface stimuli (especially in the case of AGL and SRTT) and (b) the static nature of the paradigms themselves, which seldom, if ever, afford the kinds of interactive contexts that nevertheless constitute the core feature of social life.

To overcome these limitations, we developed a novel task consisting of a learning phase and of an awareness test phase. In the learning phase, based on the classic DSC task, participants responded to the cinematic facial expressions of a realistic avatar, with the aim of bringing its facial expression in a predefined state. Unbeknownst to the participants, their responses were related to the avatar’s expressions via a complex equation modeled after Berry and Broadbent (1984): the equation likewise took into account both the avatar’s previous expression and the participants’ response. Accordingly, the avatar’s expression was influenced by the participants’ response, while the participant had to adapt his/her response to the avatar’s previous expression; in other words, the avatar and the participant were in a continuous interactive loop throughout the learning phase. A significant increase in the number of trials in which the participants managed to bring the avatar in the target state would indicate that they have acquired knowledge from the task. The primary features of the learning phase were that participants were in a continuous interaction with the avatar and the fact that the avatar’s emotional facial expression changed in a cinematic, realistic manner—aspects that give our task an unprecedented level of external validity for studying implicit social learning.

The awareness test phase measured to what extent participants were aware of the information required to regulate the avatar’s state. As the issue of measuring awareness remains highly controversial, we discuss our choices in the following subsection.

#### The awareness measures

First, we used a subjective measure of awareness. Considering that any awareness measure requires a subjacent theory of consciousness, using a subjective measure is informed by two of the most validated empirical theories of consciousness: the global workspace theory and the higher-order theories. In brief, global workspace theories show that the information one is aware of, is globally available for the cognitive subsystems, including those responsible for verbal reporting of the information. Hence, information one is aware of should be verbally reportable (e.g., Baars, 1997; Dienes, 2012). Higher-order theories are predicated on the principle that one is aware of a mental content only to the extent that one has a meta-representation that one possesses the content (i.e., one knows that one knows something; Rosenthal, 2004). Accordingly, we assessed the existence of such higher-order representations by asking participants to express what they know about their acquired knowledge (Dienes, 2012; Dienes & Scott, 2005; Ling et al., 2018). An alternative to subjective measures is to use performance-based (so-called “objective”) methods, which equate participants’ above chance performance in tasks that require them to use certain types of knowledge, with them being aware of that knowledge. For instance, in the SRTT, if participants are able to generate a sequence similar to that they have learned, one would conclude that they are aware of the learned sequence. However, it has been found that participants are able to obtain (objectively) above chance performance, even when they (subjectively) report to have no conscious knowledge (e.g., Fu et al., 2012). Hence, above-chance objective performance can be sustained by subjectively unconscious knowledge (see e.g., Timmermans & Cleeremans, 2015, for a discussion). Of course, introspection is not perfect and subjective methods of measuring awareness have often been criticized (e.g., Berry & Dienes, 1993; Newell & Shanks, 2014: Shanks & St John, 1994; Shanks, 2010). However, most often, the object of criticism has not been their subjective character per se, but rather the manner in which they are typically administered. For example, such measures are often collected after multiple trials/responses from the participant, and hence a failure of introspection can merely reflect forgetting (see the *immediacy* criterion of Newell & Shanks, 2014). As a result, most current studies that use subjective measures attempt to ensure conditions that favor an accurate introspection: for example, participants are asked to report on their awareness after each response/trial, they are provided on each trial with written indications of what they have to report, etc. (e.g., Dienes & Scott, 2005; Jurchis et al., 2020).

Second, a proper measure of awareness needs to precisely specify what content it attempts to capture (see the *relevance* criterion of Newell & Shanks, 2014). Dienes and Scott (2005) have shown that two types of knowledge can operate in implicit learning tasks, namely structural knowledge, and judgement knowledge. Structural knowledge refers to knowledge of the learned regularities. This structural knowledge leads to the development of judgement knowledge, which refers to knowledge of whether a certain response follows or not the regularity. Thus, judgement knowledge is situationally specific and develops on the basis of more general structural knowledge. One cannot have accurate judgement knowledge (cannot know whether an item conforms or not to the learned structure) in the absence of accurate structural knowledge (i.e., without knowing something about the structure) (e.g., Dienes & Scott, 2005; Fu et al., 2012). Both judgement and structural knowledge can be either conscious or unconscious. Of primary interest for our study, as for the vast majority of implicit learning studies, was whether participants extract *accurate unconscious structural knowledge*; that is, accurate knowledge regarding the regularity, the equation, embedded in our learning phase. However, to conclude that the structural knowledge is accurate, one first has to probe that it leads to accurate judgement knowledge, or, more simply put, to accurate judgements/decisions. Hence, the standard, most practical, manner to probe the existence of accurate unconscious or conscious structural knowledge is to have participants make judgements that are based on this knowledge (to accurately determine its accuracy), while asking them to report on their subjective, conscious, access to this knowledge.

To capture the accuracy of participants’ judgement knowledge and the awareness of the underlying structural knowledge, we used two well established measures: the process-dissociation procedure (PDP; Destrebecqz & Cleeremans, 2001; Jacoby, 1991), and a subjective response attribution method (Dienes & Scott, 2005). The process dissociation method measures the accuracy of participants’ judgement knowledge by asking them to either bring or to avoid bringing the avatar in a desired state.^1^ The subjective response attribution method asks participants to judge the source of their own responses which, as detailed in the Methods section, reveals the conscious/unconscious status of their structural knowledge. Furthermore, this method also affords an assessment of the conscious/unconscious status of their judgment knowledge (i.e., whether they feel they have some confidence in their response or whether they feel they are just guessing). This method was introduced by Dienes and Scott (2005) and has become one of the most widely-used subjective methods of assessing awareness across a large variety of IL paradigms, such as the AGL (e.g., Dienes & Scott, 2005; Norman et al., 2019), the SRTT (Fu et al., 2012, 2018), evaluative conditioning (Waroquier et al., 2020), symmetry learning (Ling et al., 2018), and language learning (Paciorek & Williams, 2015). However, to our knowledge it has never been used in the DSC task.

Some previous DSC studies have found that the judgement knowledge operating in this task is accurate in the sense that, given a situation, participants know what the appropriate response should be. They also found evidence for unconscious structural knowledge in the sense that the accuracy of their judgement knowledge was independent from participants’ objective performance in a recognition task (e.g., Dienes & Fahey, 1998). Accordingly, we expect that our task, which exposes participants to a regularity that is similarly complex and, presumably, difficult to be detected consciously (cf. Jurchis et al., 2020), will also lead to the development of accurate judgement knowledge sustained by unconscious structural knowledge. We note that previous DSC studies did not assess, in a sensitive manner, the awareness of both structural and judgment knowledge. Berry and Broadbendt (1984) relied on post-experimental questionnaires that have been criticized by Shanks and St John (1994) in terms of sensitivity and relevance. Dienes and Fahey (1998) equated awareness with participants’ objective recognition performance for previous trials, not including other types of knowledge that participants could use (e.g., more abstract rules or heuristics). Saevland and Norman (2016), in a DSC task, measured participants’ confidence, which indexes only awareness of the judgment knowledge. Hence, for the first time in a DSC task, we aim to sensitively assess the conscious/unconscious status of both structural and judgment knowledge.

### Objectives and hypotheses

The main objective of this study was to determine whether IL can be involved in the acquisition of the complex regularities present in a situation involving dynamic interaction with a life-like virtual agent. Based on the previous DSC studies (e.g., Dienes & Fahey, 1998), our hypotheses were (H1) that participants will acquire the regularity, (H2) that they will possess accurate judgement knowledge, (H3) that their accurate judgement knowledge will be based both on unconscious structural knowledge and on conscious structural knowledge. The detection of accurate judgement knowledge based on *unconscious* structural knowledge would enable us to conclude that IL occurred in the task. However, compared to the existing research, our task features substantially different stimuli (dynamic facial expressions) and modes of interaction with the task (responding with a facial expression). As discussed in the previous sections, the nature of the surface stimuli and the mode of interaction with those stimuli has been shown to influence the nature and the extent of learning (e.g., Jiminez et al., 2020). Accordingly, it is entirely possible that our results may diverge from those we hypothesize and that have been found in tasks that have used non-social or less complex stimuli.

## Methods

### Participants

We determined our sample size considering the statistical power needed to test H3, because it has a smaller expected effect size than that of H1 and H2. We expected a small to medium effect size for the unconscious learning effect stipulated by H3, based on previous studies that used similar methods to measure conscious and unconscious knowledge (e.g., Fu et al., 2012, 2018). Our power analysis indicated that a one-tailed test can detect a potential difference between two paired means (i.e., within-subjects design) that has an effect size of Cohen’s *d*_*z*_ = 0.3 with a statistical power of 1 − *β* = 0.9 in a sample of 97 participants. Note that for the other hypotheses, we expected large or medium to large effect sizes (for H1, a *d*_z_ = 0.798 based on Dienes & Fahey, 1^1998^ for H2, a *η*^2^_*p*_ = 0.329, based on Fu et al., 2012; for the conscious learning effect stipulated by H3, a *d*_*z*_ = 0.67, based on Fu et al., 2012), and 97 participants provided a statistical power > 99% for all these effects. Therefore, we aimed for a sample size of at least 97, but, as participants were rewarded with partial course credit, a higher number of persons enrolled. A total of 115 first-year undergraduate students in psychology from the Babeș-Bolyai University, (99 female, *M*_age_ = 19.74, SD = 1.27) participated in this research. All participants had normal or corrected-to-normal vision, enrolled voluntarily, gave written informed consent, and were told that they could withdraw from the experiment at any time, without any negative consequences. Participants consented to have their anonymized data being made publicly available. This study respected the regulations of the Babeș-Bolyai University’s Research committee and have therefore been performed in accordance with the ethical standards laid down in the 1964 Declaration of Helsinki and its later amendments.

### Apparatus

The stimuli were designed with iClone (Version 7.2; Reallusion; 2017); the JavaScript experiment was coded in PsychoPy/PsychoJs (Peirce et al., 2019) and ran on the Pavlovia.org servers. The experiment, including all raw stimuli, is accessible for download at osf.io/q9bac. For additional information on how to access the resource package, see the Supplementary material A.

### Task and materials

In a within-group design, we used a two-step task with a learning phase and an awareness test phase. In the learning phase, participants were presented with a socially relevant environment that made it possible to quantify the on-line acquisition of knowledge. In the awareness test phase, we assessed the implicit/explicit status of the acquired knowledge.

Our learning phase is inspired by the classic DSC task (Berry & Broadbent, 1984), but brings important modifications. Amongst which the most important is the different types of surface stimuli. Specifically, on the one hand, Berry and Broadbent, as well as the bulk of the research implementing this task, used surface stimuli under the form of written words with an affective valence (e.g., “extreme anger”). On the other hand, we used dynamic facial expressions of a cinematic virtual male avatar (i.e., the video of a virtual human depicting—for instance—an actual, standardized, facial expression of extreme anger). The reason for which we decided not to design a female avatar is informed by the AGL study of Ziori and Dienes (2015), who found that the perceived attractiveness of stimuli depicting female faces affects learning performance of both male and female participants. Given that this effect did not appear for stimuli depicting male faces neither for male nor for female participants, we prevented the potential stimulus attractiveness effects on IL by designing socio-emotionally relevant surface stimuli that were expressed by a male avatar. In the next subsection, we detail the characteristics of these emotional facial expressions.

#### The emotional facial expressions

**Fig. 1:**
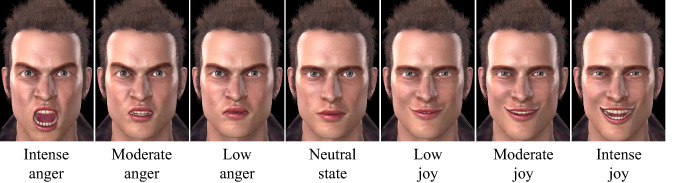
The seven facial expressions used in this study

In our task, participants interacted with a cinematic virtual avatar that could display a range of seven emotional facial expressions (see Fig. 1) and transitioned from one facial expression to the next in a fluid motion comprised in fixed intervals of 30 frames, lasting 500 ms.

The contraction amplitudes and movement successions of all digital muscles (i.e., Action Units, AUs) were linear and respected the guidelines provided by the Facial Action Coding System (FACS; Ekman et al., 2002). If on two trials, the avatar had to morph between two levels of the same emotional facial expression (e.g., from Low anger to Intense anger), the transition was programmed to occur simply by increasing the contraction amplitude of the relevant AUs; instead, if on two trials, the avatar had to morph between facial expressions of different families, the transition was programed to occur by passing through the Neutral state.^2^ The last fame of the 30-frame transition—i.e., the frame with the peak amplitude—remained on the screen for the entire duration of the trial. Next, we clarify the manner in which the transitions between different facial expressions were sequenced to create the learning phase.

#### The learning phase

Participants were informed that they will interact with a fictional character from an unknown culture who is able to display only a limited number of facial expressions and is unable to regulate his facial expressions. They were further instructed that the avatar attends an important task in which he must not express intense facial expressions—neither positive nor negative—and that their task is to assist him in regulating his emotions, aiming to get him into the Neutral state as many times as possible. Then, participants were instructed on how they could interact with the avatar. Specifically, they were told that the avatar will display a facial expression and they will have to show him a picture of himself that they think will bring him in the Neutral emotional state. For instance, if the avatar displays Intense anger, they will have to show him a picture of himself that they think can calm him down to the Neutral state. All trials in which participants succeeded to regulate the avatar in the target (i.e., Neutral) state were classified as *On-target* trials and were considered as the index of learning. Lastly, participants were told that because the avatar is from this unknown culture, he may react to the picture that they show him in ways that are not necessarily normal or typical. In sum, in the learning phase, on each trial, participants (1) saw a facial expression of the avatar and (2) had to respond by selecting an expression, so that the avatar’s expression would change to neutral in the next trial (or remain neutral, if the expression was already neutral).

Crucially, undisclosed to participants, in the first milliseconds of each trial, the program determined the facial expression that the avatar will morph into by computing a rule that took into account both the avatar’s expression in the previous trial and the participant’s response in the previous trial. We detail the contents of this rule in the subsection below.

##### The abstract rule

**Fig. 2:**
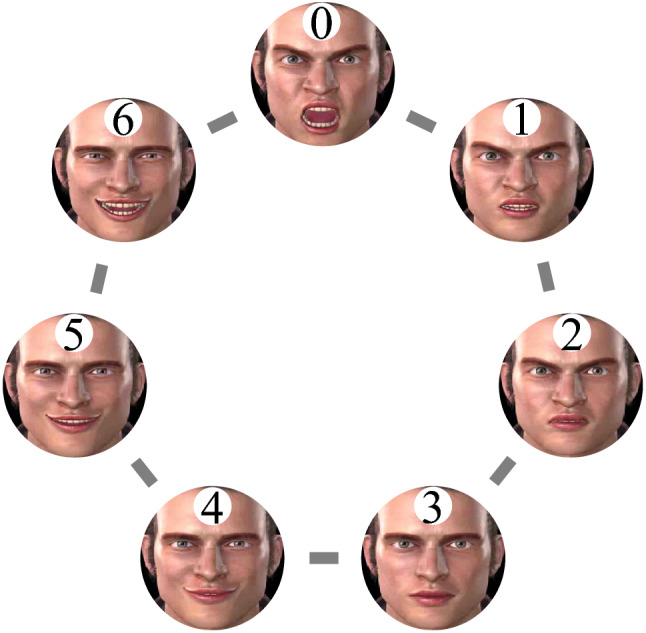
The abstract looped numerical sequence*.* It depicts both the avatar’s possible facial expressions and the participants’ possible response options. Intense anger = position 0, moderate anger = position 1, low anger = position 2, the neutral state = position 3, low joy = position 4, moderate joy = position 5 and, intense joy = position 6

To describe our implementation of the equation, it is first necessary to present the fact that each of the avatar’s 7 possible facial expressions, as well as each of the participants’ seven possible response options were assigned a constant position within a looped numerical sequence; for a graphical representation, see Fig. 2. The starting point of the sequence was set on position 0 (i.e., Intense anger) however, transitions within the sequence could be made in both a clockwise or an anticlockwise direction. Participants were not directly exposed to, or made aware of, the existence of this sequence.

To determine the avatar’s facial expression on any given trial (i.e., Av. Exp*.*_*t*_), the task was programmed to compute the equation “Av. Exp._*t*_ = 0 + [Av. Exp._*t*−1_ + (Av. Exp._*t*−1_ − P. Resp._*t*−1_)]” where, “Av. Exp._*t*_” denotes the Avatar’s expression in the current trial; “0” represents the starting point of the stimulus set (i.e., the facial expression of Intense anger; this parameter remained constant from trial to trial, throughout the task); “Av. Exp._*t*−1_” represents the Avatar’s expression in the previous trial and “P. Resp._*t*−1_” represents the Participant’s response in the previous trial.

The result of the equation indicated the direction and length of the pathway that the task moved within the looped sequence—starting from position 0—to select the avatar’s facial expression in the current trial. The task was programed to move within the sequence in a clockwise direction if the result was a positive number and vice versa if the result was a negative number. Participants were instructed to regulate the avatar’s facial expression in the Neutral state as many times as possible. Thus, all instances in which “Av.Exp._*t*_ = 0 + 3” or, “Av.Exp._*t*_ = 0–4” were considered *On-target trials* because, according to these results, the avatar will morph into the Neutral facial expression. For a possible interaction sequence between a participant and the task across the first three trials of a block, see the Table 1 below (for a complete matrix that contains all the possible interactions, see the Supplementary material B).

**Table 1:** Simulates how the equation mediates the interaction between a participant’s responses and the avatar’s facial expressions

Event order	Event description	Equation
1	Avatar’s expression in trial 1 is I*ntense anger*	Av. Exp._*t*1_ = 0 [i.e., Intense anger]
2	If participants’ response in trial 1 is N*eutral*	P. Resp._*t*1_ = 3 [i.e., Neutral]
3	Computation of the required change in position	Change_*t*2_ = 0 + [Av. Exp._*t*1_ + (Av. Exp._*t*1_ − P. Rresp._*t*1_)] Change_*t*2_ = 0 + [0 + (0 − 3)] Change_*t*2_ = 0 + [0 + (− 3)] Change_*t*2_ = 0 + (− 3)
4	The location moves three positions counterclockwise, starting from zero	Change_*t*2_ = − 3
5	**Outcome**: Avatar’s expression in trial 2 is *low joy*	Av. Exp._*t*2_ = 4 [i.e., Low joy]
6	If participants’ response in trial 2 is *intense joy*	P. Resp._*t*2_ = 6 [i.e., Intense joy]
7	Computation of the new state by the algorithm	Change_*t*3_ = 0 + [Av. Exp._*t*2_ + (Av. Exp._t2_ − P. Resp._*t*2_)] Change_*t*3_ = 0 + [4 + (4 − 6)] Change_*t*3_ = 0 + [4 + (− 2)] Change_*t*3_ = 0 + 2
8	The location moves two positions clockwise, starting from zero	Change_*t*3_ = + 2
9	**Outcome**: Avatar’s expression in trial 3 is *low anger*	Av. Exp._*t*3_ = 2 [i.e., low anger]
10	If participants’ response in trial 3 is *moderate anger*	P. Resp._*t*3_ = 1 [moderate anger]
11	Computation of the new state by the algorithm	Change_*t*4_ = 0 + [Av. Exp._*t*3_ + (Av. Exp._t3_ − P. Resp._*t*3_)] Change_*t*4_ = 0 + [2 + (2 − 1)] Change_*t*4_ = 0 + [2 + (1)] Change_*t*4_ = 0 + 3
12	The location moves three positions clockwise, starting from zero	Change_*t*4_ = + 3
13	**Outcome**: Avatar’s expression in trial 4 is *Neutral*	Av. Exp._*t*4_ = 3 [i.e., Neutral]

The table describes the events from three consecutive trials. Av. Exp._*tx*_—the avatar’s expression in trial *x*. P. Resp._*tx*_—participants’ response in trial *x*; Change_*tx*_—the change in position within the looped sequence, required for reaching the avatar’s state in trial *x*

Noteworthy, our task has no specific input—specific output mapping; therefore, task habituation cannot explain performance improvements. Instead, participants could become proficient in controlling the system by engaging in an abstract learning process or, alternatively, by engaging in an instance-specific learning process. To better exemplify, it is possible that participants acquired the abstract equation from the task {i.e., Av.Exp._*t*_ = 0 + [Av.Exp._*t*−1_ + (Av.Exp._*t*−1_ − P.Resp._*t*−1_)]} or, alternatively, it is possible that they eventually learned to respond adequately in several specific interaction instances [e.g., “If the avatar’s expression is Moderate anger and I select a response of Intense anger then its expression becomes Neutral.” or “If the avatar’s expression is Neutral, and I select a response of Neutral, then its expression remains Neutral”]. Note that, according to the equation, after the avatar reached the Neutral state, it could be maintained in this state indefinitely simply by repeatedly choosing the Neutral response option. However, to prevent participants from obtaining a high performance in the learning phase based on this instance-specific rule, they were not allowed to repeat the same response in two consecutive trials. In this case if the avatar reached the Neutral state, participants could maintain its expression Neutral for just another trial (i.e., by selecting the response of Neutral). If they attempted to repeat their response, a feedback message was displayed on the screen and participants were asked to select a different response.

Participants interacted with the avatar dynamically. Specifically, given the same facial expression of the avatar, two different responses from the participants would produce different responses from the avatar in the next trial (for an example, view the Supplementary material C).

Task performance was indexed by the number of trials in which participants regulated the avatar to the Neutral facial expression (i.e., *On-target trial*). In other words, an *On-target trial* was achieved when the result of the equation indicated the facial expression that will be displayed by the avatar is the Neutral state (i.e., Av. Exp. = 0 + 3 or, Av. Exp. = 0–4). Evidence of learning was considered if the number of *On-target trials* increased with practice (i.e., as the task progressed). In the next section, we present a specific account of the events that occurred during a typical trial.

##### A typical trial structure

**Fig. 3:**
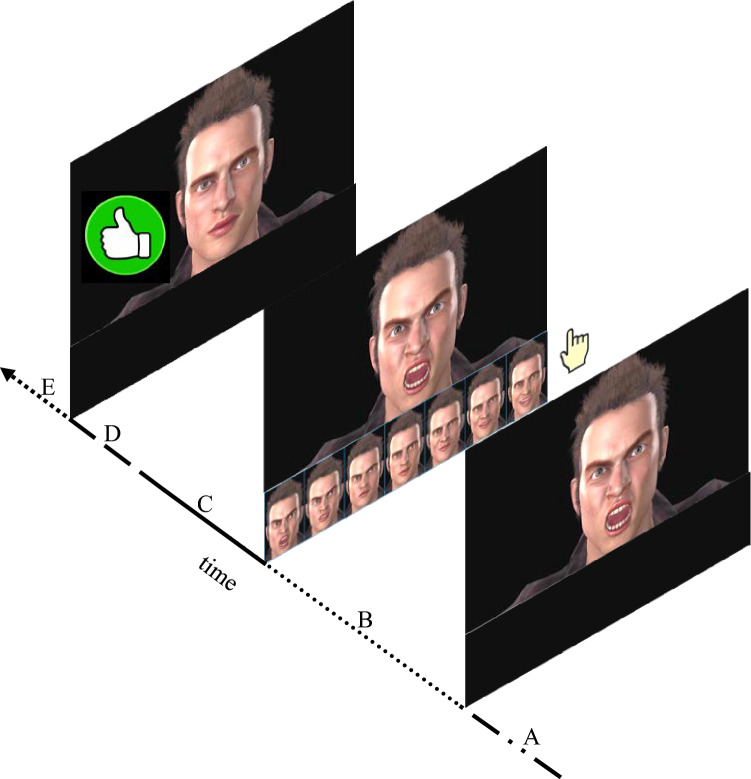
Depicts a typical trial structure. *A* = 500 ms. (This is the time interval in which the task first, determined the facial expression that will present in the current trial, based on both, the avatar’s expression and participant’s response in the earlier trial and second, morphed the facial expression of the avatar from the expression that it displayed in the earlier trial to the one that it will display in the current trial); *B* = 500 ms. (The time interval in which only the facial expression of the avatar at peak amplitude was displayed on the screen); *C* = 6000 ms. (The seven facial expressions which served as response options were displayed horizontally on the bottom of the screen. After a response has been made, the experiment went ahead to the next trial.); *D* = 2999 ms. (The counter appeared on the screen if no response has yet been given); *E* = ∞ (The late response feedback appeared on the screen if no responses were registered)

At the beginning of each block, the avatar started a preprogramed transition from the Neutral expression to a facial expression of Intense anger. This transition occurred as presented in the Sect. 2.3.1. The transition between the two emotional facial expressions elapsed over a time interval of 500 ms (see the segment A of Fig. 3). The last frame of the transition (i.e., Intense anger at peak amplitude) remained exposed on the screen until a response from the participant was given, the program computed the next facial expression of the avatar and the transition/morphing of the avatar toward that facial expression started. After the transition was complete, for a time-interval of 500 ms the facial expression at peak amplitude remained the only stimulus being exposed on the screen. This time interval was introduced to encourage active processing of the current facial expression (see the segment B of Fig. 3). Then, the seven facial expressions which served as response options were displayed horizontally on the bottom of the screen for a time interval of maximum 10 s (see the segments C and D of Fig. 3). Participants were asked to click on the facial expression with which they wish to respond. If participants did not indicate a response after 7 s, a counter appeared on the screen. This counter asked them to respond in no more than three seconds. (See the segment D of Fig. 3). If a response had not been registered, a “*late response*” feedback was presented on the screen. The trial was marked as “late” in our data bases and it was discarded from the analyses (see the segment E of Fig. 3). If a response has been made within the 10 s interval, the program moved to the next trial. The “thumbs-up” feedback appeared on the left side of the screen only on those trials where the avatar reached the target, Neutral state (see Fig. 3). If participants repeated more than two consecutive responses, a “*repeated response, chose another!*” feedback was displayed on the screen, participants were asked to select a different response, and the specific trial was discarded from analyses.

The learning phase was structured as a self-paced seven alternative forced choice task (7AFC) and consisted of 300 trials divided to ten equal blocks, with 30 s rest breaks between each of them.

#### The awareness test phase

The main goal of this phase was to determine whether participants had acquired accurate unconscious and conscious knowledge about the equation (structural knowledge). To this end, we employed an extensively used subjective measure of awareness, called *knowledge attribution* (e.g., Dienes & Scott, 2005; Fu et al., 2012, 2018; Norman et al., 2011, 2016, 2019; Waroquier et al., 2020) which was used in the context of a *Process Dissociation Procedure* (PDP; Destrebecqz & Cleeremans, 2001; Jacoby, 1991). In the following, we will present the specific modality in which we combined these two measures in the present experiment.

The PDP assesses participants’ ability to flexibly operate with their acquired knowledge. The procedure consists of two tasks: inclusion and exclusion. As applied here, in each trial of both tasks, participants were presented with an image depicting one of the previously encountered facial expressions of the avatar. They were then asked to respond (by selecting a facial expression out of the same response options that were used in the learning phase) to either bring the avatar in the target state (in the inclusion task) or to bring the avatar in any other state but the target state (in the exclusion task). The avatar’s seven facial expressions were randomly presented twice in each task. The task order effect was controlled by a counterbalanced design, 64 participants started the test phase by completing the inclusion task, and the other 51 participants started the test phase by completing the exclusion task. To prevent learning during the test phase, the experiment proceeded without feedback as soon as a response has been given. If participants regulated the avatar to the target state on significantly more trials in inclusion vs. exclusion, we would conclude that they had acquired accurate judgement knowledge. Crucially, however, accurate judgement knowledge can be based on unconscious structural knowledge (e.g., I feel that the fourth facial expression would bring the avatar in the target state, but I have no idea why). Indeed, this is what was found by most—if not all—IL studies that used a PDP in conjunction with subjective measures: participants are able to control their knowledge even when they are subjectively unaware of the learned structures (e.g., Fu et al., 2012, 2018; Norman et al., 2011, 2016, 2019). Accordingly, to examine the accuracy of judgement knowledge and, also, to uncover the conscious/unconscious status of structural knowledge, we employed this mixed assessment of awareness, which combines the PDP with knowledge attributions.

Participants were required to indicate the subjective attribution of their response after each trial of the PDP. Do participants think that the response they just produced (in the PDP) reflects conscious knowledge of the *rules* or *memory* of a similar instance? Did they respond based on an *intuition* they cannot justify? Or did they feel that they just *guessed*? The response attribution trial was presented as four alternative forced choice (4AFC) with *Guess*, *Intuition*, *Rules* and *Memory* as response options. The *Guess* and *Intuition* response options denote that participants attribute their answer to unconscious structural knowledge (hereinafter *implicit attributions)* whereas the *Rules* and *Memory* response options denote that participants attribute their answer to conscious structural knowledge (hereinafter, *explicit attributions*). Participants were presented with explanations of these response options after each trial of the PDP (see Table 2 below) and were asked to choose the option that they think best describes what they relied on when they gave the previous answer.

**Table 2:** Definition of the self-reported decision strategies

Guess	Your answer had no basis whatsoever. You could have just as well flipped a coin to decide
Intuition	You felt that your answer was correct but you have no idea why you felt this. That is, you had a feeling that by responding with that facial expression, you were regulating John in the Neutral state—but you do not know what that impression was based on
Rules	Your answer was based on a rule (or on a fragment of a rule) that you know consciously and you can describe if we ask
Memory	Your answer was based on the fact that you consciously remember that by responding with that facial expression you were bringing John in the Neutral state

The existence of accurate unconscious structural knowledge is inferred if, in trials in which participants use implicit attributions (i.e., *Guess* and *Intuition*), they are able to accurately use their judgement knowledge (i.e., by including significantly more responses that conform to the learned equation in inclusion than in exclusion; cf., e.g., Fu et al., 2012, 2018).In the following section, we present the specific sequence of instructions and tasks that were administered to the participants.

### Procedure

Next, we will present an overview of our procedure—for a more detailed account, consult the Supplementary material D. Participants were first asked to give written informed consent prior to completing the experimental activities. Second, they were asked for their demographic information. Third, they completed the learning phase and, fourth, they completed the awareness test phase. The entire experiment lasted around 25 min.

In the learning phase, before initiating the experiment, participants were given the following written instruction: “You will interact with John, a fictional character from an unknown culture; he is able to display only a limited number of facial expressions and is unable to regulate his facial expressions in the way that we do. John attends an important task and in order to be successful, he must not express intense facial expressions—neither positive nor negative. Your task is to assist him in regulating his facial expressions, aiming to bring him into a neutral state as many times as possible. Finally, John may respond to your inputs in ways that might seem atypical”. After participants read the instructions, we presented the feedback messages that could appear in this phase of the experiment (i.e., the positive feedback, the feedback for repeated responses and the feedback for late responses). Before initiating the experimental trials of the learning phase, we verified that participants knew how to interact with the avatar by asking them to perform 10 practice trials. Here, the avatar reacted randomly to participants’ inputs and these trials were dropped from the analyses.

After they completed the learning phase, participants were given the written instructions for the awareness test phase. Participants were informed that they would be presented with a facial expression of the avatar and they would have to choose the response they thought would bring the avatar in the target state (for the inclusion task) or in any other state but the target (for the exclusion task). They were further instructed to indicate the subjective basis of their response by choosing one of the four possible response options (i.e., Guess, Intuition, Rules or Memory). After participants indicated that they understood their task, we presented them with one practice trial (from the inclusion or the exclusion phase, depending on the condition). The practice trial was also discarded from the analyses. After completing the practice trial, participants started the awareness test phase. The definition of the response options appeared on the screen after each trial of the PDP.

After the awareness test phase was completed, participants were thanked for their involvement in this research and were given the contact information of the principal investigator in order to address their potential questions.

## Results

In the following, we will first analyze whether participants acquired knowledge of the regularity. Then, we analyze whether they possess accurate judgement knowledge. Last, we assess whether their accurate judgement knowledge is based both on unconscious structural knowledge and on conscious structural knowledge.

### (*H1*) Did learning occur?

**Fig. 4:**
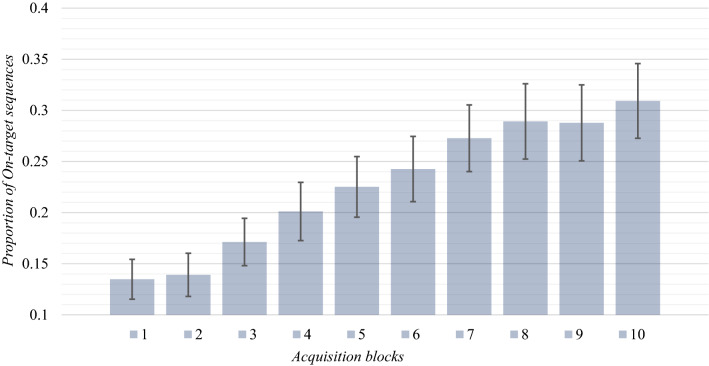
The mean proportion of On-target trials (i.e., the trials in which participants managed to bring the emotional facial expression of the avatar in the Neutral state) generated across the acquisition blocks. Error bars depict 95% CIs

The raw dataset generated for this study is available on the Center for Open Science repository (osf.io/q9bac). If participants acquired knowledge from the task, we would expect an increase of the number of *On-target trials* as the task progressed. A one-way repeated measures ANOVA revealed a significant effect of *Block* (1–10, within-subjects) on the number of *On-target trials, F*(9, 114) = 38.33, *p* < 0.001, *η*^2^_*p*_ = 0.252. A follow up repeated measures *t* test indicated that participants generated significantly more *On-target trials* in the *10th acquisition block* (*M*_proportion_ = 0.309, SD = 0.198) than in the *1st acquisition block* (*M*_prop*.*_ = 0.135, SD = 0.106), *t*(114) = − 9.05, *p* < 0.001, Cohen’s* d* = 0.84. Altogether, these results clearly show that learning had occurred during the task (see Fig. 4).

### (*H2*) Did participants possess accurate judgement knowledge?

Participants’ ability to flexibly conform to the opposing requirements of the inclusion and of the exclusion tasks would be enabled by their possession of accurate judgement knowledge. To determine whether they possessed this accurate judgement knowledge, we compared the number of *On-target trials* from the inclusion task with those from the exclusion task*.*

A mixed ANOVA assessed the effects of *Instruction* (within-subjects: inclusion vs. exclusion) and *task order* (between-subjects: inclusion–exclusion vs. exclusion–inclusion) on the number of *On-target trials* generated in the test phase. We found a significant *Instruction* effect, *F*(1, 113) = 107.01, *p* < 0.001, *η*^2^_*p*_ = 0.486, indicating that participants generated significantly more *On-target trials* in the inclusion (*M*_prop._ = 0.357, SD = 0.220) than the exclusion (*M*_prop._ = 0.104, SD = 0.098) task. We failed to detect either a significant *Task order* effect, *F*(1, 113) = 0.61, *p* = 0.435, *η*^2^_*p*_ = 0.005, or a significant *Instruction* by *Task order* interaction effect *F*(1, 113) = 0.07, *p* = 0.794, *η*^2^_*p*_ = 0.005. These results suggest that participants acquired accurate judgement knowledge from the task. For a graphical representation and analysis of participants’ accuracy in the PDP against the chance level, consult Supplementary material E.

### (*H3*) Did participants acquire accurate conscious and unconscious structural knowledge?

After having established that participants possessed accurate judgement knowledge, we now analyze to what extent it is based on unconscious and/or on conscious structural knowledge.

Following established analytical strategies in IL research (Norman & Price, 2012; Ziori & Dienes, 2015), we combined the Guess and Intuition response attributions to create *Implicit attributions* scores, and the Rules and Memory attributions to create the *Explicit attributions* scores. Roughly half of the total responses were based on *Implicit attributions* (in inclusion, *M*_prop._ = 0.528, SD = 0.306 and in exclusion, *M*_prop._ = 0.465, SD = 0.368) and the other half were based on *Explicit attributions* (in inclusion, *M*_prop._ = 0.472, SD = 0.306 and in exclusion, *M*_prop._ = 0.535, SD = 0.368). For a detailed perspective on the distribution for each of the four response bases (i.e., Guess, Intuition, Rule and Memory) across the inclusion and the exclusion tasks of the PDP, see the Table 3 below.

**Table 3:** The frequency of each response basis options within the two PDP tasks

Guess		Intuition		Rule		Memory	
Inclusion	Exclusion	Inclusion	Exclusion	Inclusion	Exclusion	Inclusion	Exclusion
0.263 (0.246)	0.173 (0.236)	0.265 (0.227)	0.292 (0.299)	0.260 (0.292)	0.291 (0.348)	0.212 (0.257)	0.244 (0.307)

The values represent proportions and, in paratheses, standard deviations.

We then analyzed the accuracy of participants’ judgement knowledge depending on the conscious/unconscious status of their structural knowledge. First, we assessed whether participants had accurate judgement knowledge when they reported that their responses were based on explicit structural knowledge. Note that the overall analysis only included the 96 (out of 115) participants, who reported relying on an *explicit attribution* (i.e., Rule or Memory) on at least one trial in both the inclusion and the exclusion tasks. For responses based on *Explicit attributions*, a paired sample *t* test indicated that participants generated significantly more *On-target* trials in the inclusion (*M*_prop._ = 0.558, SD = 0.347) than the exclusion task (*M*_prop._ = 0.072, SD = 0.114), *t*(96) = 13.02, *p* < 0.001, *d* = 1.32. In a more granular perspective, for responses based on *Rules*, a paired sample *t* test indicated that participants generated significantly more *On-target trials* in the inclusion (*M*_prop._ = 0.542, SD = 0.369) than the exclusion task (*M*_prop._ = 0.062, SD = 0.133), *t*(62) = 9.38, *p* < 0.001, *d* = 1.18. The same pattern of results was observed for the responses based on *Memory*—participants generated significantly more *On-target trials* in the inclusion (*M*_prop._ = 0.537, SD = 0.401) than in the exclusion task (*M*_prop._ = 0.076, SD = 0.159), *t*(53) = 10.09, *p* < 0.001, *d* = 1.37. Collectively, the analyses above indicate that participants had accurate judgement knowledge on trials where they reported relying on conscious structural knowledge.

We then assessed whether participants had accurate judgement knowledge when they reported that their responses were based on unconscious structural knowledge. Note that the overall analysis only included the 87 (out of 115) participants who reported relying on an implicit attribution (i.e., Guess or Intuition) in at least one trial in both the inclusion task and the exclusion tasks. For responses based on *Implicit attributions*, a paired sample *t* test indicated that participants generated significantly more *On-target trials* in the inclusion (*M*_prop._ = 0.200, SD = 0.209) than the exclusion task (*M*_prop._ = 0.130, SD = 0.156), *t*(87) = 2.88, *p* = 0.006, *d*_*z*_ = 0.31. In a more granular perspective, for responses based on *Intuition*, a paired sample *t* test indicated that participants generated significantly more *On-target trials* in the inclusion (*M*_prop._ = 0.186, SD = 0.229) than the exclusion task (*M*_prop._ = 0.126, SD = 0.153), *t*(73) = 1.67, *p* = 0.05, *d* = 0.19. Furthermore, even for responses that were attributed to *Guess*, the inclusion (*M*_prop._ = 0.210, SD = 0.287)—exclusion (*M*_prop._ = 0.134, SD = 0.222) difference on the number of *On-target trials* that were generated by the participants remained statistically significant, *t*(61) = 1.76, *p* = 0.041, *d* = 0.224.

In the following, we employed an alternative method to verify if IL occurred in the task: the guessing criterion (Dienes et al., 1995)—which can be computed by comparing participants’ performance in the inclusion task against the chance level. However, to do this, it was first necessary to specify our chance level. Given that in each trial of the PDP, out of the 7 possible response options, only one could regulate the avatar’s facial expression to the target state, our chance level is 0.143. According to the guessing criterion, if participants acquired unconscious knowledge of the rule, we would expect them to show above-chance performance for the trials on which they indicated that they based their responses on guesses. Indeed, congruent with the analysis above, a one-sample *t* test indicated that even when indicating guessing as a response attribution, participants generated significantly more *On-target trials* (*M*_prop._ = 0.201, SD = 0.287) than what would be expected at the chance level (i.e., 0.143), *t*(88) = 2.26, *p* = 0.0013, Cohen’s *d* = 0.239.

Collectively, the analyses above clearly indicate that participants had accurate judgement knowledge on trials where they reported relying on unconscious structural knowledge—when they reported basing their answers on an intuition or, even when they indicated that they had chosen them at random.

## Discussion

We aimed to investigate whether it is possible to learn and use, without awareness, regularities that govern interactions with a virtual avatar that displays socio-emotional content, under the form of dynamic facial emotional expressions. The results are largely consistent with our hypotheses and with other evidence obtained in previous IL studies that have used the DSC paradigm (Berry & Broadbent, 1984). Namely, our data indicate that learning occurred, demonstrated by the fact that participants gradually increased their ability to regulate the avatar’s facial expression to the target state during the learning phase. Furthermore, they were also able to flexibly operate with the acquired knowledge in the PDP, even when their self-report indicated that they were relying on guesses and intuitions, that is, when they felt they were not using conscious knowledge of the rules. Instead, according to the terminology of Scott and Dienes (2005), they relied on unconscious knowledge of the structure their responses were based on (see also Fu et al., 2012, 2018; Norman et al., 2011, 2016, 2019). This indicates that, indeed, participants have extracted unconscious knowledge of the equation governing our task. Nevertheless, participants acquired a consistent amount of accurate conscious knowledge indicated by the fact that, in the PDP, they could operate accurately with their knowledge, when they reported awareness of it. As the issue of awareness can be controversial, in the next subsection we would like to discuss several aspects of our findings with respect to the involvement of unconscious knowledge in our task.

### The conscious: unconscious status of learning

First, people frequently (i.e., in 49.65% of the trials, averaged over inclusion and exclusion tasks) reported that their decisions were based on implicit decision strategies, indicating that they did not feel that they were aware of the structure that guided their responses in the PDP. We note that initial IL studies operated on the assumption that an accurate PDP performance indicates conscious knowledge of the learned structure; that is, participants would be able to respond according to the learned structure in inclusion, or avoid responding according to it in exclusion, only if they were aware of the structure (e.g., Destrebecqz & Cleeremans, 2001). Nevertheless, a plethora of more recent studies have shown that equating PDP performance with awareness is unwarranted, since subjectively unconscious knowledge can successfully guide participants’ PDP performance. To our knowledge, this is what every study that has supplemented the PDP with subjective response attribution has found (Fu et al., 2012, 2018; Norman et al., 2011, 2016, 2019; Wan et al., 2008), and that is what we have also found: that participants can flexibly follow the requirements of the inclusion and of the exclusion tasks, even when they report not being aware of the structure. Our results also conceptually replicate previous DSC research; similar to Fahey and Dienes (1998), we found that the judgement knowledge is accurate in the sense that, given a situation, participants know what the appropriate response should be. Furthermore, similar to Fahey and Dienes, we found evidence for unconscious knowledge in the sense that subjective access to the memory representation enabling accurate judgement is not required for optimal task performance. More to this point, we consider that this pattern of results resembles natural social phenomena; for instance, situations in which one knows how to behave in a certain context even if one is unable to consciously access the underlying knowledge which substantiated one’s decision/way of action.

Second, as in most other IL tasks, our results do not provide a clear answer regarding the exact nature of the acquired knowledge. For instance, as discussed in the introduction, in the AGL task, participants could learn both specific combinations of letters that make a string grammatical or not, but could also learn more abstract types of knowledge pertaining to the structure of the grammar (such as the global or the local repetition proportion; see, e.g., Scott & Dienes, 2008). Similarly, in the context of DSC, Dienes and Fahey (1995) have found that participants’ performance is not typically guided by the abstract equation embedded in the task, but rather by implicit and explicit memories of interacting with the system on specific trials. Accordingly, we claim that participants have extracted structural knowledge from the task, because they had accurate performance in the PDP and in turn, accuracy in the PDP requires at least knowledge of the mappings between stimuli and responses. However, we do not claim that participants necessarily developed an abstract, mathematical, representation of the equation itself. Rather, it is likely that they have acquired a memory base regarding specific interactions with the task and their outcome. This memory base then informed participants’ ability to conform to the opposing tasks of the PDP while being partly implicit (i.e., the proportion of *On-target* trials attributed to implicit response bases was 0.198 in the inclusion and 0.13 in the exclusion) and partly explicit (i.e., the proportion of *On-target* trials attributed to explicit response bases was 0.542 in the inclusion and 0.07 in the exclusion task).

A third aspect regarding the conscious/unconscious status of learning in the present study is that our task has produced a higher proportion of trials attributed to explicit decision strategies compared to the already established IL paradigms. For example, most AGL studies reveal that about 2/3 of the responses are attributed to unconscious structural knowledge (e.g., Dienes & Scott, 2005). In our study, participants attributed their responses to strategies based on unconscious structural knowledge on only about half the trials (see Table 3 above). Thus, it seems that our participants felt that they were applying explicit knowledge to a larger extent than participants have done in other IL experiments. This apparent enhanced acquisition of explicit knowledge in our task affords several candidate explanations. According to the first these candidate accounts, as in all DSC studies, our instructions asked participants to control the state of a system—this instruction by itself is likely to have triggered a more analytical processing style (Dienes & Scott, 2005). A second explanation might be that, in contrast to previous DSC research, we did not use noise in our task. Specifically, in each trial of Berry and Broadbent’s (1984) acquisition phase, the novel state of the system was determined by running an equation to which the computer added 1, 0, or − 1 units on a random basis. Crucially, responses were correct if they were either on target or one unit around the target. We consider that this liberal method to quantify task performance has the potential to be contaminated by both false-positives as well as false-negatives. Therefore, we chose not to introduce the random factor.^3^ As a third explanation, previous research argued that different implementations of the PDP offer different estimates of the implicit and explicit judgement knowledge. For instance, in the context of the SRTT, by using a PDP with free generation tasks, Destrebecqz and Cleeremans (2001) showed that participants acquired unconscious judgement knowledge of a sequence; whereas by using a PDP with cued generation tasks, Wilkinson and Shanks (2004) indicated that participants acquired explicit judgement knowledge of a sequence. Our version of the PDP used pseudo-cued generation tasks. Specifically, instead of being exposed to a succession of stimuli, participants were exposed to only one facial expression and asked to generate the next to either bring the avatar in the target state (in the inclusion task) or avoid bringing the avatar in the target state (in the exclusion task). As Stahl et al. (2015) showed that the cued generation tasks have the potential to introduce bias in the PD estimates, future research could investigate if the implicit-explicit estimates of the acquired knowledge from our paradigm differ as a function of the generation task (free generation vs cued generation).

### Contributions

The discovery of implicit learning for rules that govern dynamic interactions with a social stimulus advances the existing tools for investigating the involvement of nonconscious learning in social contexts in several important ways, which we will detail in the paragraphs below.

First, as briefly mentioned in the introduction, results obtained in the hidden covariation detection task indicated that participants were able to implicitly learn an arbitrary covariation between a physical feature and personality features, and that the judgement of new avatars is influenced by the learned covariation (Ivanchei et al., 2019; Lewicki, 1986). On a more distal note, Geiger et al. (2018) have shown that social cues (the gaze of an avatar) can guide and facilitate the development of implicit knowledge in a motor task (a variant of the SRTT). While they employed surface stimuli that are relevant for social functioning, the loops through which information is being exchanged between participants and these tasks of these studies are fundamentally different from those in which information is exchanged in social environments. Specifically, while in these tasks (i.e., Geiger et al., 2018; Ivanchei et al., 2019; Lewicki, 1986) information is being exchanged via noninteractive loops, in the real social environment, information is being exchanged via feedback-driven interactive loops. The fact that our task implements such feedback-driven interactive loops increases its external validity for evaluating the function of IL in the social domain.

Second, the present study is the first one to use surface stimuli under the form of realistic emotional facial expressions. The inclusion of socially relevant surface stimuli in our task does not have a mere cosmetic effect but is motivated by two arguments: a first argument, as presented in the introduction, is that the existing studies show that IL is highly sensitive to the stimuli it operates on: the same regularity can be learned to a variable degree (e.g., Jimenez et al., 2020) or can be learned more or less explicitly (Norman & Price, 2012) depending on the characteristics of the surface stimuli. To our knowledge, this is the first study to use kinematic, realistic, facial expressions, bringing our research one step closer to the habitual mode of processing this information in the real social environment. A second argument for the necessity of tailoring the experimental task to the domain of investigation (e.g., social, motor, linguistic) derives from a core principle of current neural and computational theories of IL: that is, IL occurs through changes in the speed, strategy, and efficiency of processing specific stimuli with different processing constraints. For instance, in their influential paper, Frost et al. (2015) present ample evidence that implicit and statistical learning are represented by “a set of domain-general computational principles that operate in different modalities and, therefore, are subject to the specific constraints characteristic of their respective brain regions” (see also Conway, 2020; Reber, 2013).

Third, the strategy for assessing knowledge awareness represents another strong point of the study. Specifically, previous DSC research measured knowledge awareness by direct questioning (i.e., post experimental questionnaires) or verbal report. To the best of our knowledge, this is the first DSC study to employ trial by trial subjective measures of awareness (i.e., response attributions and the PDP); thereby, offering a more precise perspective on the way in which explicit knowledge, as well as implicit knowledge, contribute to task performance.

### Limitations and implications for future research

First, the test phase consisted of precisely the same interaction instances encountered in the acquisition phase. Therefore, on the grounds of this method, we cannot infer transfer of the learned information to novel situations. Future research could use different avatars across the acquisition blocks while maintaining the same rule that structures the interactions to assess the transference of the learned content to novel situations. At this stage, our task cannot provide a definitive answer to whether participants learned the abstract mathematical equation or they learned specific interaction sequences (Dienes & Fahey, 1995). Third, the interaction between participants and the avatar was centered around a limited number of emotional facial expressions that were displayed in exactly the same parameters—however, in the natural social environment, different configurations of a facial expression can express, for instance, an intense level of joy; these two aspects depart from the diversity that characterizes genuine social environments. Hence, future research could employ a larger variety of different surface stimuli that would express similar emotional states.

Despite the importance of IL for social functioning, investigations focusing on the IL of socially relevant information are relatively limited. A potential cause for this may be represented by the challenges involved in the development of methodologically sound experimental paradigms that assess IL in a socially relevant context. Given that the implicit acquisition of regularities unfolds differently depending on the characteristics of the stimuli that follow those regularities (e.g., on their perceptual complexity, motivational relevance, habitual mode of processing those stimuli; see, e.g., Jimenez et al., 2020; Seger, 1994), we suggest that the inclusion of socially relevant surface stimuli could enable our task to reveal untapped, social, facets of this non-homogenous process. We conclude by suggesting that our findings could foster novel research avenues into the role that IL might play in different conditions characterized by atypical social functioning. For instance, it could complement or even replace, in some cases, the AGL and SRTT in studies that investigate the integrity of implicit learning in autism spectrum disorders, depression etc.

## Conclusions

The present study is one of the first to propose a task for assessing the role of IL in interactive situations with socially relevant surface stimuli. Furthermore, by employing one of the most versatile measures of awareness that was used in the DSC research up until this point, we provide evidence that, similar with other well-established IL tasks (e.g., the AGL and SRTT), our DSC task indeed produces implicit knowledge, along with a significant amount of explicit knowledge.”

## Supplementary Information

Below is the link to the electronic supplementary material.

Supplementary file1 (DOCX 182 KB)

## Data Availability

Data are available at osf.io/q9bac.

## Footnotes

1 We note here that methods derived from Jacoby’s (1991) PDP were initially used in IL research for revealing the conscious/unconscious status of learning. They assumed that, if participants can flexibly follow the requirements of the inclusion and exclusion tasks, then they are aware of the learned knowledge (e.g., Destrebecqz & Cleeremans, 2001). More recent studies, however, have shown that accurate PDP performance can be based on unconscious structural knowledge (Fu et al., 2012, 2018; Norman et al., 2011, 2016, 2019; Wan et al., 2008), and even on unconscious judgement knowledge (Norman et al., 2019). Accordingly, we consider it unwarranted to take accurate PDP performance as an indicator of awareness. Instead, we use it only for establishing the accuracy of participants’ judgement knowledge. As explained, to determine the conscious/unconscious character of knowledge we use subjective measures.

2 For instance, if between two trials, the avatar had to morph from a facial expression of Intense anger to one of Moderate joy then, the transition initially started by decreasing the intensity of the facial expression of Intense anger towards the Neutral state (this process elapsed over the first 14 screen refreshes of the trial); after reaching the Neutral state (where it remained for two screen refreshes), the program then increased the expressiveness of the avatar from the Neutral state to reach the facial expression of Moderate joy (this process elapsed over another 14 screen refreshes).

3 Future research could design a more complex equation that would promote the acquisition of rather implicit than explicit knowledge or, alternatively, implement a shorter acquisition phase.

## Code availability

The experimental task is available for download at https://gitlab.pavlovia.org/AndreiRazvanCostea/implicit_social_learning_task.
