# Supplementary material for: Implicit and explicit learning of socio-emotional information in a dynamic interaction with a virtual avatar
Source: Psychol Res. 2022 Aug 29;87(4):1057–74. doi: 10.1007/s00426-022-01709-4 (PMC10191928; doi:10.1007/s00426-022-01709-4)
Supplement: Supplementary file 1 — Supplementary file1 (DOCX 182 KB) [file 426_2022_1709_MOESM1_ESM.docx]

# Supplementary Material of the article

Implicit and explicit learning of socio-emotional information

in a dynamic interaction with a virtual avatar

As submitted to the journal Psychological research

Andrei R. Costea^a,b*^, Răzvan Jurchiș^a^, Laura Visu-Petra^c^,

Axel Cleeremans^d^, Elisbeth Norman^e^, and Adrian Opre^a^

^a^ Cognitive Psychology Laboratory, Department of Psychology, Babeș-Bolyai University, Cluj-Napoca, Romania

^b^ Department of Socio-Human Research, Romanian Academy, Cluj-Napoca Branch, Romania

^c^ Research in Individual Differences and Legal Psychology (RIDDLE) Laboratory, Department of Psychology, Babeș-Bolyai University, Cluj-Napoca, Romania

^d^ Consciousness, Cognition & Computation Group (CO3), Center for Research in Cognition & Neuroscience (CRCN), ULB Neuroscience Institute (UNI), Université Libre de Bruxelles, Bruxelles, Belgium

^e^ Department of Psychosocial Science, Faculty of Psychology, University of Bergen, Bergen, Norway

*** Corresponding author:**Name: Andrei R. Costea
E-mail address: [andreicostea@psychology.ro](mailto:andreicostea@psychology.ro)

## Supplementary material A


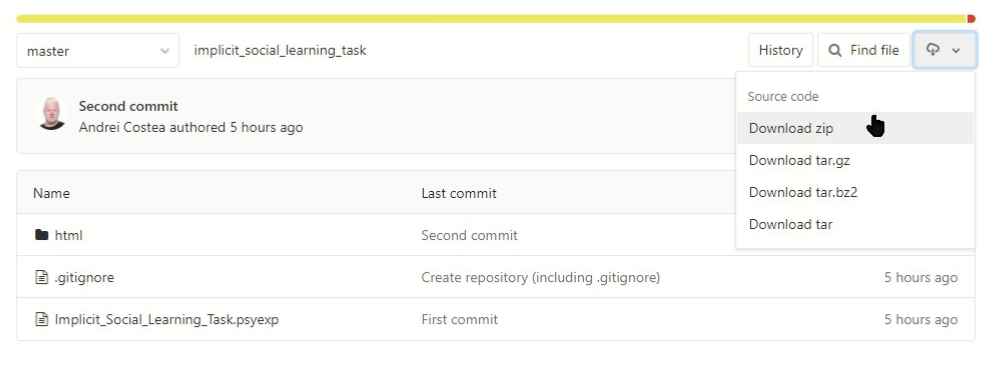


**Supplementary Fig 1.** *Depicts a partial screenshot from the GitLab/Pavlovia server, where the behavioral task is stored and illustrates how to download it as a .zip fie. The server contains the PsychoJs script along with all stimuli used in the experiment at https://gitlab.pavlovia.org/AndreiRazvanCostea/implicit_social_learning_task.*

## Supplementary material B

|  |  | Participant’s response in the trial n-1 | | | | | | |
| --- | --- | --- | --- | --- | --- | --- | --- | --- |
|  |  | Intense anger | Moderate anger | Low  anger | Neutral state | Low  joy | Moderate joy | Intense  joy |
| Avatar’s expression in trial n-1 | Intense  Anger | Intense anger | Intense  joy | Moderate joy | Low  joy | Neutral state | Low  anger | Moderate anger |
|  | Moderate  Anger | Low  anger | Moderate anger | Intense anger | Intense  joy | Moderate joy | Low  joy | Neutral state |
|  | Low  Anger | Low  joy | Neutral state | Low  anger | Moderate anger | Intense anger | Intense  joy | Moderate joy |
|  | Neutral  State | Intense  joy | Moderate joy | Low  joy | Neutral state | Low  anger | Moderate anger | Intense anger |
|  | Low  Joy | Moderate anger | Intense anger | Intense  joy | Moderate joy | Low  joy | Neutral state | Low  anger |
|  | Moderate  Joy | Neutral state | Low  anger | Moderate anger | Intense anger | Intense  joy | Moderate joy | Low  joy |
|  | Intense  Joy | Moderate joy | Low  joy | Neutral state | Low  anger | Moderate anger | Intense anger | Intense  joy |

**Note.** *The table cells present the result of the equation in trial n, given all possible participant’s responses to all possible avatar’s states in trial n-1.*

## Supplementary material C

In this example, we simulate the effect of two different responses from the participants, given the same previous facial expression of the avatar. Suppose that the in the 10^th^ trial of a block, the avatar displayed a facial expression of Intense anger. One participant (i.e., Participant A) attempted to bring the avatar in the target state by selecting the Neutral response option (i.e., *P. Resp._t10_* = 3) while another participant (i.e., Participant B) attempted to bring the avatar in the target state by selecting the Moderate anger response (i.e., P. Rresp._t10_ = 1). The effects of these different responses are presented in the Supplementary table 1 below.

Supplementary table 2. Effects of two different participant responses on the same previous expression of the avatar

| Participant A | Participant B |
| --- | --- |
| *Av. Exp._t11_ = 0 + [Av. Exp._t10_ + (Av. Exp._t10_ - P. Resp._t10_)]*  *Av. Exp._t11_* = 0 + [0 + (0 – 3)]  *Av. Exp._t11_* = 0 + [0 + (– 3)]  *Av. Exp._t11_* = 0 + (-3)  *Av. Exp._t11_* = Low joy | *Av. Exp._t11_ = 0 + [Av. Exp._t10_ + (Av. Exp._t10_ - P. Resp._t10_)]*  *Av. Exp._t11_* = 0 + [0 + (0 – 1)]  *Av. Exp._t11_* = 0 + [0 + (-1)]  *Av. Exp._t11_* = 0 + (-1)  *Av. Exp._t11_* = Intense joy |

## Supplementary material D

Participants were first asked to give their electronic written informed consent. Second, they were asked for their demographic information. Third, they received the instructions for the learning phase. Fourth, they completed a ten trials training block of the learning phase. Fifth, they completed the experimental learning phase. Sixth, they received the instructions for the awareness test phase. Finally, they completed the awareness test phase. The entire experiment lasted around 25 minutes. In the following paragraphs we present a nuanced account of the entire experimental procedure.

In the first screen of the instructional phase, participants were presented with a picture of the avatar in the Neutral facial expression. Above the avatar, they were presented with the following instructional text: In this task, “*you will interact with John, a fictional character from an unknown culture. He can display only a limited number of facial expressions and is unable to regulate his facial expressions in the way that we do. John attends an important task and to be successful, he must not express intense facial expressions - neither positive nor negative”.* Under the picture of the avatar, the following text appeared: “*To see the facial expressions that can be expressed by John, press the space key*”.

In the second instructional screen, participants were presented with the following text: “*John comes from a strange culture - which treats facial expressions differently than we do - and he can only express different levels of anger and joy (see the figure below). Additionally, John does not know to regulate his facial expressions*”. Below this text, on the centre of the screen, we presented the both, the avatar’s 7 possible emotional facial expressions and their corresponding names (similar with the Figure 1). In the lower section of this screen, participants were exposed to the following text: “*Given that, on the one hand, John has an important task to do - in which he must reach the Neutral state as many times as possible and, on the other hand, he is unable to regulate his emotions, your task is to assist him in regulating his emotions. In brief, you need to help John get into the Neutral emotional state as many times as possible*”. On the bottom of this screen, a button stated: “*To see how you can do this, press the space key*”.

In the third instructional screen participants were presented with the following test: “*John will first show you a facial expression (figure A) and you will have to show him a picture of himself that you think will bring him in the Neutral emotional state. For example, if John is terribly angry, you will have to choose a picture of himself that you think can calm him down to the Neutral state. Importantly, because he is from this unknown culture, John may react to the picture that you show him in ways that are not necessarily normal or typical for us. You will have to choose your answer from Figure B below. You will answer by clicking on the white circle that appears above the expression you want to show to John”*. In the centre of this instructional screen, we presented the two figures: Figure A, depicting John in a static facial expression of Intense anger, and Figure B, depicting the 7 possible response options. On the bottom of this screen, a button stated: “*To see how such an interaction works, press the Space key”.*

In the training block, the avatar started the interaction by displaying a randomly chosen facial expression to which the participants were required to respond as described above. After a response from the participants has been registered, the avatar proceeded to the next trial by presenting another randomly chosen facial expression, etc. The training block lasted for 10 trials; differently than the experimental learning phase, no time limit was set for the training trials.

After they completed the training session, in the fourth instructional screen, participants were presented with the following text: “*We observe that you know how to interact with John! You did very well! You are almost ready to start the experiment. However, until we begin, there are a couple of rules that you need to know, look at the table below*”. In the centre of this instructional screen, we presented participants with a visual, depicting each feedback sign/message that they could encounter along the learning phase (see table 3 below).

**Table 3.** *Definition of the feedback messages that were encountered by the participants*

| Positive feedback  *(a thumbs-up sign)* | When you bring John in the Neutral state, this sign will appear on the left side of the screen |
| --- | --- |
| Repeated response  *(a red circle with the text ‘**repeated response, chose another’ written in white)* | You are not allowed to use the same answer twice in a row. In other words, you are not allowed to repeat two consecutive answers. If this happens, this sign will appear on the right side of the screen and you will be asked to choose another answer. |
| Countdown timer  *(a 3-seconds countdown timer)* | Try not to think too much about each answer. We advise you to respond in less than 10 seconds. After 7 seconds have elapsed since we will display the response options, this countdown will appear on the right side of the screen. When the countdown reaches 0, a ‘*slow response’* message will appear. You will be able to answer even then, but that answer will not be considered. |

At the bottom of this instructional screen, a button stated: “*To begin the experiment, press the space key*” and another button stated “*to look at all the instructions again, press the Esc key*”. The task progressed to the learning phase after participants pressed the space key.

The learning phase consisted of 300 trials, which were divided in 10 equal blocks. A reast break of 30 seconds occurred after each block. During each break, participants were presented with a text which summarised their task progress (i.e., “*You completed 10% of the task. We continue in: 30, 29, 28, … 0 Seconds”*).

After they had completed the learning phase, participants were given the written instructions for the awareness test phase. Participants were informed that they would be presented with a facial expression of the avatar and they would have to choose the response they thought would bring the avatar in the target state (for the inclusion task) or in any other state but the target (for the exclusion task). They were further instructed to indicate the subjective basis of their response by choosing one of the four possible response options (*Guess*, *Intuition*, *Rules*, *Memory*). A back translated version of the definitions that we used is presented in Table 2. After participants indicated that they understood their task, we presented them with one practice trial (from the inclusion or the exclusion phase, depending on the condition in which they were randomly assigned in). Nevertheless, the practice trial was discarded from the analyses. After completing the practice trial, participants started the awareness test phase. The definition of the response options appeared on the screen after each trial of the PDP. After the awareness test phase was completed, participants were thanked for their involvement in this research and were given the contact information of the principal investigator to address their potential questions.

## Supplementary material E

To determine the chance level, we take in consideration the fact that in each trial of the PDP, out of the 7 possible responses, only one could regulate the avatar’s facial expression to the target state. Since our generation tasks (i.e., inclusion and exclusion) had 14 trials each, we expected that participants will generate at least 2 *On-target trials* in each task at chance level (i.e., *M_prop._* = .142). If participants indeed had accurate judgement knowledge, we expect to find that they generated a proportion of *On-target trials* significantly larger than the one expected at the chance level in the inclusion task and significantly smaller than the one expected at the chance level in the exclusion task. For the inclusion task, a one-sample *t* test indicated that participants generated a proportion of *On-target trials* significantly larger than the proportion expected at the chance level *M_prop._* = .357, *SD* = .220, *t*(114) = 10.54, *p* < .001, *d* = 0.973. For the exclusion task, a one-sample *t* test indicated that participants generated a proportion of *On-target trials* significantly smaller than the proportion expected at the chance level *M_prop._* = .105, *SD* = .098, *t*(114) = 4.139, *p* < .001, *d* = 0.386. Together these results provide additional evidence to the claim that participants developed accurate judgement knowledge. For a graphical representation, see the Supplementary Figure 2.

**Supplementary Fig 2** *The mean proportion of On-target trials that were generated by the participants in the inclusion and exclusion tasks of the PDP against the chance level. Error bars represent 95% CIs.*
